# Antarctic Toothfish *Dissostichus mawsoni* in the South Orkney Islands: Using Otolith Chemistry to Test Current Hypotheses About Nursery Areas and Demographic Units

**DOI:** 10.3390/biology14010007

**Published:** 2024-12-25

**Authors:** Paulina Carimán, Edwin J. Niklitschek, Cristóbal Garcés, Mathieu Leisen, Fernando Barra, Rurik Romero

**Affiliations:** 1 Institute of Environmental and Evolutionary Sciences, Faculty of Sciences, Austral University of Chile, Valdivia 5091000, Chile; pj.cariman@gmail.com; 2 i~mar Centre, University of Los Lagos, Puerto Montt 5480000, Chile; 3 Fishing Research Program, Austral University of Chile-University of Los Lagos, Puerto Montt 5480000, Chile; 4 Doctoral Program in Ecology and Evolution, Faculty of Sciences, Austral University of Chile, Valdivia 5091000, Chile; garcesbima@gmail.com; 5 Geosciences-Environment Toulouse, University of Toulouse, 31400 Toulouse, France; mleisen@ing.uchile.cl; 6 Andean Geothermal Center of Excellence, University of Chile, Santiago 8370446, Chile; fbarrapantoja@ing.uchile.cl (F.B.); rurikrom@gmail.com (R.R.)

**Keywords:** Antarctic toothfish, nursery areas, demographic units, otolith chemistry, stock identification, stable isotopes

## Abstract

We used otolith chemistry to test and complement current hypotheses regarding habitat use and connectivity between *Dissostichus mawsoni* sub-populations in Area 48 of the Commission for the Conservation of Antarctic Marine Living Resources (CCAMLR). Sagittal otoliths from 45 fish sampled near the South Orkney Islands were analysed. Their elemental (Li, Na, Mg, Cr, Mn, Sr, Sn, and Ba relative to Ca) and isotopic (δ^18^O and δ^13^C) signatures were examined in both the nuclear and marginal regions, representing juvenile and adult stages. Potential nursery habitats were geo-located by comparing observed and expected δ^18^O values. Chemical differences between the nuclear and marginal regions indicated ontogenetic migrations to deeper offshore habitats, suggesting a distinct habitat shift between 11 and 13 years of life. The data supported the existence of two nursery origins contributing to the study area’s population. However, the exact locations of these origins remain unclear and did not provide direct support for the hypotheses currently under consideration by the CCAMLR. Therefore, further assessment of the connectivity between nursery and adult habitats, as well as spawning site fidelity, is necessary before ruling out alternative hypotheses.

## Simple Summary

In this study, we investigated the habitat use of the Antarctic toothfish (*Dissostichus mawsoni*) and the connectivity between nursery and feeding areas in the Atlantic sector of the Southern Ocean. We analysed the chemical signatures of otoliths (ear stones) from 45 individuals caught near the South Orkney Islands. These structures provide insights into the fish’s life stages, from juvenile to adult. By examining the chemical composition of the otoliths, we identified potential nursery areas where juvenile fish develop. Our findings suggest that Antarctic toothfish migrate to deeper waters at approximately 11 to 13 years of life and that two distinct nursery areas contribute to the population in this region, supporting one of the hypotheses regarding its habitat use. However, further research is needed to fully understand the connections between breeding and nursery sites before excluding other potential explanations for their habitat use. This study is crucial for effective population management and the development of sustainable fishing practices in the Southern Ocean.

## 1. Introduction

The Antarctic toothfish *Dissostichus mawsoni* is a benthopelagic member of the family Nototheniidae, which has a circumpolar distribution, primarily south of 57° S and closely associated with the Antarctic Convergence [1,2]. With a median age and size at first maturity of 13–16 years and ~100 cm total length (TL), its lifespan reaches 50 years, exceeding 200 cm TL and 100 kg of individual mass [3,4]. Several ontogenetic habitat shifts occur during this long lifespan [5,6]. While young-of-the-year (YOY) and yearlings (4–12 cm TL) use shallow pelagic waters close to the coast, older juveniles (19–54 cm TL) become demersal and spend a variable number of years on the continental shelf at depths between 50 and 300 m [7,8,9]. Subadults (50–95 cm TL) then move to greater depths (500–850 m) on the continental slope [10]. After reaching sexual maturity (~100 cm TL), adults become neutrally buoyant [5,11,12], and expand their bathymetric range greatly, feeding between 10 and 2200 m, although showing some preference for depths between 300 and 500 m [2,13,14].

**Figure 1:**
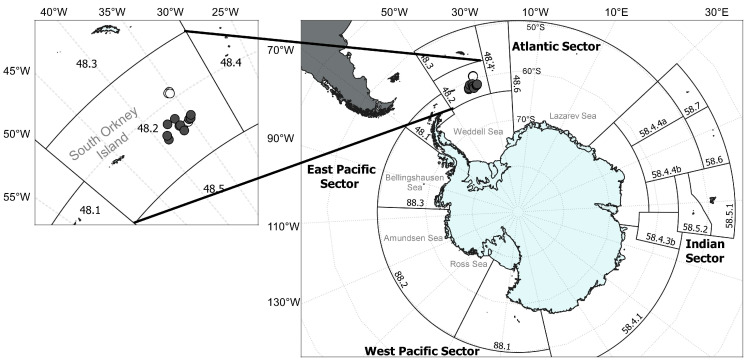
**Right panel** shows the Antarctic coastline, including the areas, sub-areas, and Small-Scale Research Units defined by the CCAMLR (Convention for the Conservation of Antarctic Marine Living Resources). **Left panel** shows the fishing area and sampling locations west of the South Orkney Islands, where filled and empty circles represent the distribution of fish caught in 2016 and 2017, respectively.

*Dissostichus mawsoni* supports one of the two most valuable commercial fisheries in the Southern Ocean, largely in international waters managed by the Commission for the Conservation of Antarctic Marine Living Resources (CCAMLR). The CCAMLR’s jurisdiction is divided into areas, sub-areas, and Small-Scale Research Units (SSRUs; Figure 1), which are expected to share relatively similar oceanographic and biological characteristics and to contain relatively discrete populations of some target species [15]. For some others, these CCAMLR divisions may not represent biologically relevant (evolutionary or demographic) units, leading to potentially important mismatches between biology and management actions [16,17] In the case of *D. mawsoni*, both the number and distribution of evolutionary and demographic units across the Southern Ocean and the degree of matching between these units and CCAMLR areas or sub-areas are poorly understood. This uncertainty and incomplete knowledge about the structure, connectivity, and dynamics of *D. mawsoni* demographic units have become core issues in recent criticisms of CCAMLR regulations, which are considered too risky given that several key parameters of the stock assessment model [18], estimated almost exclusively from biological and fishery data from sub-area 88.1, remain unknown or largely uncertain [19].

The limited knowledge of *D. mawsoni* evolutionary units results not only from the small number of studies conducted to date but also from variability in sampling designs, study areas, and molecular techniques [20,21,22]. Tagging studies have shown that while most marked adults remained for years within 50 km of their tagging sites, a few others moved up to 2300 km away [7,23]. Despite its low frequency, these movements could be enough to obscure or counteract ongoing genetic differentiation processes across extensive areas [24], biasing the estimation of demographic units, as well as leading to the actual existence of a rather small number of evolutionary units. Nonetheless, the dominance of resident behaviour suggests the existence of separate *D. mawsoni* demographic units, isolated enough to exhibit asynchronous population dynamics and different life-history parameters [16]

Despite its practical relevance for management purposes, uncovering the number, distribution, and connectivity between *D. mawsoni* demographic units (i.e., stocks) has received little attention so far [23,25,26]. These studies, primarily focused on sub-areas 88.1 and 88.2, which concentrate most of the current fishing activity, have shaped the current working hypothesis that a single demographic unit exists in these two sub-areas [7,27], while a second demographic unit is thought to exist west of the Antarctic Peninsula [25,26]. No studies have been explicitly devoted to scrutinizing the structure of demographic units within other areas or sub-areas. This includes Area 48, which is located to the east of the Antarctic Peninsula and encompasses the Weddell Sea and part of the Lazarev Sea (Figure 1). This area represented 14% of the fishery catch in 2018; however, given evidence of overexploitation for several stocks at that time, most of it has remained closed to all finfish fisheries since 1990 [28].

While fishery-dependent data are scarce after years of fishing closure, fishery-independent information is also quite limited for *D. mawsoni* in Area 48 [29]. Nonetheless, after major efforts, these authors have proposed three main hypotheses about the structure and connectivity of *D. mawsoni* demographic units in this area: H1, a single demographic unit exhibiting some limited connectivity with area 58 (Indian Ocean sector); H2, two separate units, both placed within the Wedell Sea, with origins in sub-areas 48.2 and 48.6; and H3, at least three interconnected demographic units, with origins in sub-areas 48.1, 48.2, and 48.6 and some limited connectivity with area 58.

The aim of the present work was to scrutinize Söffker et al.’s hypotheses [29], leveraging insights provided through otolith chemistry methods to fish samples collected around the South Orkney Island (sub-area 48.2) during exceptional exploratory activities authorized in 2016 and 2018. Otolith chemistry has proven useful for identifying and determining demographic units, as well as migration and habitat use patterns, in multiple species across different temporal scales when sufficient variability exists between habitats occupied by different groups. In this particular application, we utilized observed variability in elemental and isotopic compositions of otolith nuclear regions to estimate the most informative number of nursery origins and applied isotopic-based geo-location techniques [30,31,32] to map the likely origin of the sampled fish across sub-population areas depicted by CCAMLR working hypotheses.

## 2. Materials and Methods

### 2.1. Sampling

The sagittal otoliths of 45 adult *D. mawsoni* were collected around the South Orkney Islands (Figure 1) during two exploratory fishing trips, authorized exceptionally to be conducted by the Chilean FV Cabo de Hornos in the closed sub-area 48.2, during 2016 and 2018. The first set of 21 samples was collected across 8 different tows, between 25 February and 3 March 2016. A second set of 24 samples was collected from another 8 tows, between 4 February and 12 February 2018. After fish age and cohort (birth year) were estimated through the inverse function of the von Bertalanffy growth model reported by Horn [4], six fish born before 1989 were discarded to reduce somewhat interannual variability. Thus, a subset of 39 fish born between 1989 and 2002 were retained for further analysis. This subset included 24 females, 14 males, and one unsexed individual. Total length ranged between 122 and 166 cm, while total weight ranged between 22 and 55 kg.

### 2.2. Chemical Composition of Otoliths

**Figure 2:**
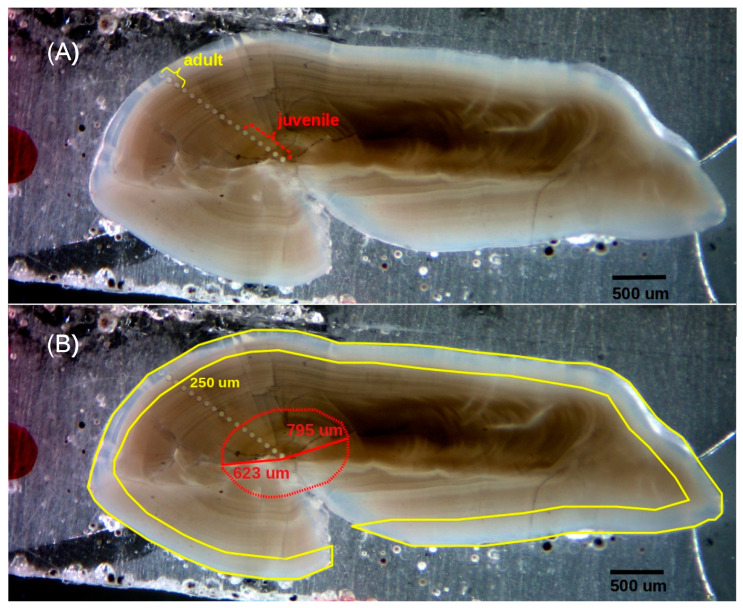
Transverse section of a sagittal otolith from an adult *Dissostichus mawsoni* specimen (>135 cm TL), polished and analysed by LA-ICPMS. (**A**) A total of 17 ablations (diameter = 65 μm) are observed along the primordial–ventral margin axis of the otolith. The first six ablations represent the juvenile stage, while the last three correspond to the adult stage. (**B**) Micro-drill trajectory programmed to extract nuclear (red polygon) and marginal (yellow polygon) regions of the sagittal otolith for isotopic composition analysis.

Otoliths were extracted, rinsed with seawater, dried with a paper towel, and stored in paper bags at sea. Once in the laboratory, they were rinsed again using ultrapure water (18.2 MΩ), photographed, sonicated in ultrapure water for 5 min, and dried under a laminar flow hood, following clean protocols using clean plastic forceps and disposable gloves. Otoliths were then embedded in high-purity epoxy resin (EpoxiCure^TM^, Buehler; Lake Bluff, IL, USA) and cut (low-speed ISOMET Buehler saw) on both sides of the primordium to obtain transverse sections of 450–900 μm. Sections were first polished with progressively finer silicon carbide cloths (1500, 2000, and 2500 grit) down to a thickness of 200–400 μm (Figure 2), and then with 60 μm and 3 μm diamond lapping film (Buehler) to expose primordia and remove contamination from silicon carbide cloths. Once polished, sections were sonicated for 5 min in ultrapure water, dried under a laminar flow hood, mounted on slides using thermoplastic glue (Crystalbond™, Ted Pella Inc.; Redding, CA, USA), and stored for further analyses.

### 2.3. Elemental Composition Sampling and Analysis

Elemental composition sampling and analysis were carried out by a 193 nm ArF laser ablation (LA) system (Photon Machines Analyte G2, Teledyne CETAC Technologies; Omaha, NE, USA) coupled to an iCapQ ThermoScientific ICP-MS (Thermo Fisher Scientific; Waltham, MA, USA). Ablation was performed following a radial transect of spots, from the primordium to the ventral or, if not possible, dorsal edge of the section (Figure 2A). Distance between ablations was accommodated slightly (93–97 μm) to cover the whole transect, yielding 14–24 ablations per otolith, depending on its size. Given a fluence of 5 J·cm^−2^, a frequency of 10 Hz, and a period of 30 s, each ablation reached a depth of 20–30 μm, with an average diameter of 65 μm. Silicate glass reference material NIST SRM 612 (NIST; Gaithersburg, MD, USA) was used as the primary external standard [33], while the USGS synthetic calcium carbonate MACS-3 [34] was employed for quality control. Ca was used as the internal standard, assuming a stoichiometric value of 38.8% for CaO in the aragonite matrix [35]. Ablation points within this region with elevated Al concentrations were excluded from the analysis to avoid potential contamination.

External standard measurements were performed before and after ablating each otolith. Data generated by the mass spectrometer were reduced by using Iolite V2.5 [36] “Trace Elements IS” reduction scheme. In total, concentrations of 38 elements (40 mass numbers) were quantified: ^7^Li, ^11^B, ^23^Na, ^24^Mg, ^25^Mg, ^27^Al, ^31^P, ^52^Cr, ^55^Mn, ^57^Fe, ^59^Co, ^60^Ni, ^63^Cu, ^66^Zn, ^75^As, ^83^Kr, ^85^Rb, ^86^Sr, ^88^Sr, ^89^Y, ^111^Cd, ^120^Sn, ^121^Sb, ^125^Te, ^133^Cs, ^138^Ba, ^139^La, ^140^Ce, ^141^Pr, ^146^Nd, ^147^Sm, ^169^Tm, ^174^Yb, ^175^Lu, ^178^Hf, ^206^Pb, ^207^Pb, ^208^Pb, ^232^Th, and ^238^U. However, only eight elements exhibited concentrations above detection limits in ≥95% of the samples and were selected for further analysis: Li, Na, Mg, Cr, Mn, Sr, Sn, and Ba.

Although LA transects extended from the core to the edge of each otolith section, we focused, for most analyses, on the nuclear and marginal regions (Figure 2A). The nuclear region included the 2nd to 6th ablations (93–540 μm from the core) and was set to represent most of the first year of life, based on reported measurements of 623–795 μm for the first annulus radius [8]. The first ablation was purposely excluded to minimize potential maternal effects. The edge region included the last three ablation points (~255 μm) from each LA transect, representing the last 5–7 years of life. No finer resolution was possible here, given slow otolith accretion rates in adult *D. mawsoni* and minimum material requirements for isotopic analysis (~30 µg). Ablation points within this region with elevated Al concentrations were excluded from the analysis to prevent potential contamination.

### 2.4. Stable Isotope Analysis

Powder samples (≥30 μg) from nuclear and marginal regions, again, designed to represent juvenile (0–540 μm from the core) and adult life stages (255 μm from the edge), were obtained using a New Wave Research microdrill equipped with a 500 μm diameter bit (Figure 2B). Whenever possible, the same otoliths and sections were used for both elemental composition and stable isotope analyses to maintain consistency. The extracted material was analysed at the University of Arizona Environmental Isotope Geochemistry Laboratory using an automated KIEL-III carbonate preparation device coupled to a Finnigan MAT 252 ratio-gas spectrometer (Thermo Finnigan; Bremen, Germany). Digestion was undertaken in vacuum-dehydrated phosphoric acid at 70 °C, with the CO_2_ generated by this reaction used to determine δ^18^O and δ^13^C values (in parts per thousand) relative to Vienna Pee Dee Belemnite (VPDB). To do so, international standards NBS-19 and NBS-18 were used as primary and secondary standards. In practice, δ_VPDB_ values were computed as:

δ*X*_VPDB_ = (δ*X_SAMPLE/_*_NBS-19_ + 1) × (δ*X*_NBS-19/VPDB_ + 1) − 1

where δ*X_SAMPLE/_*_NBS-19_ = 1000 × (1 − *R_SAMPLE_/R_NBS-19_*), *X* represents either ^13^C or ^18^O, *R* is the isotopic ratio measured in the mass spectrometer, and δ*X*_NBS-19/VPDB_ is the nominal δ value of NBS-19 relative to VPDB: 1.95‰ for δ^13^C and −2.20‰ for δ^18^O [37].

### 2.5. Statistical Analysis

As a general approach, we purposely avoided any dichotomic use of *p*-values, following recommendations from the American Statistical Association [38] and an increasing number of independent scientists [39]; we instead favoured confronting alternative models against the available data [40,41], as well as the presentation of effect sizes, standard errors, and related probabilities, whenever possible. Further details provided for each goal are as follows.

#### 2.5.1. Confronting Ontogenetic Habitat Shift Hypotheses Against Otolith Chemistry Data

Elemental and isotopic signatures of nuclear and marginal regions were compared using univariate and multivariate techniques. Univariate analyses followed a mixed-effects linear modelling approach [42], as implemented in the R package nlme v3.1.165 [43]. Multivariate analyses were based on a permutational multivariate analysis of variance (PERMANOVA), as implemented in the R package vegan v2.6.4 [44], using Mahalanobis distance metrics. Residual quantile–quantile (QQ) plots showed no severe departures from univariate and multivariate normal distributions. Consistency with univariate and multivariate homoscedasticity assumptions was verified using Levene’s [45] and Anderson’s tests [46]. The lack of independence expected between observations from the same fishing event was accounted for by incorporating this variable as a random factor in univariate models and as a fixed one in multivariate ones.

Sr:Ca y Ba:Ca ratios showed the greatest variability both between and within our fish samples. Since these two ratios are known to reflect environmental variability in temperature, salinity, or freshwater inflows [47,48,49], we used them to identify and date discrete habitat shifts experienced by each sampled fish along its ontogeny. Habitat shift probability was estimated for each ablation along the variability within individuals, along the LA-ICPMS profile, using the Bayesian point change method of Barry and Hartigan [50], as implemented in the R package bcp v4.0.3 [51], running 10,000 simulations, discarding the first 5000 as burn-in, and using a prior value of 0.2 for both the signal/noise ratio and the probability of shift detection.

#### 2.5.2. Estimating the Number and Mixing Between Nursery Areas

We fitted a series of finite mixture distribution (FMD) models [52], defined by a variable number of 1–5 sources, to either elemental or isotopic signatures observed in otolith nuclear regions. Source contributions, means, and covariance matrix parameters for each source were estimated using the expectation-maximization (EM) algorithm [53], as implemented in the R package mclust v6.0.1, restricted by us to consider only uneven-volume covariance models [54]. Model selection was performed using Schwarz’s Bayesian Information Criteria [55], following the general approach of Niklitschek and Darnaude [56].

#### 2.5.3. Geo-Locating Nursery Areas to Confront Söffker et al. (2018)’s Hypotheses [29]

**Figure 3:**
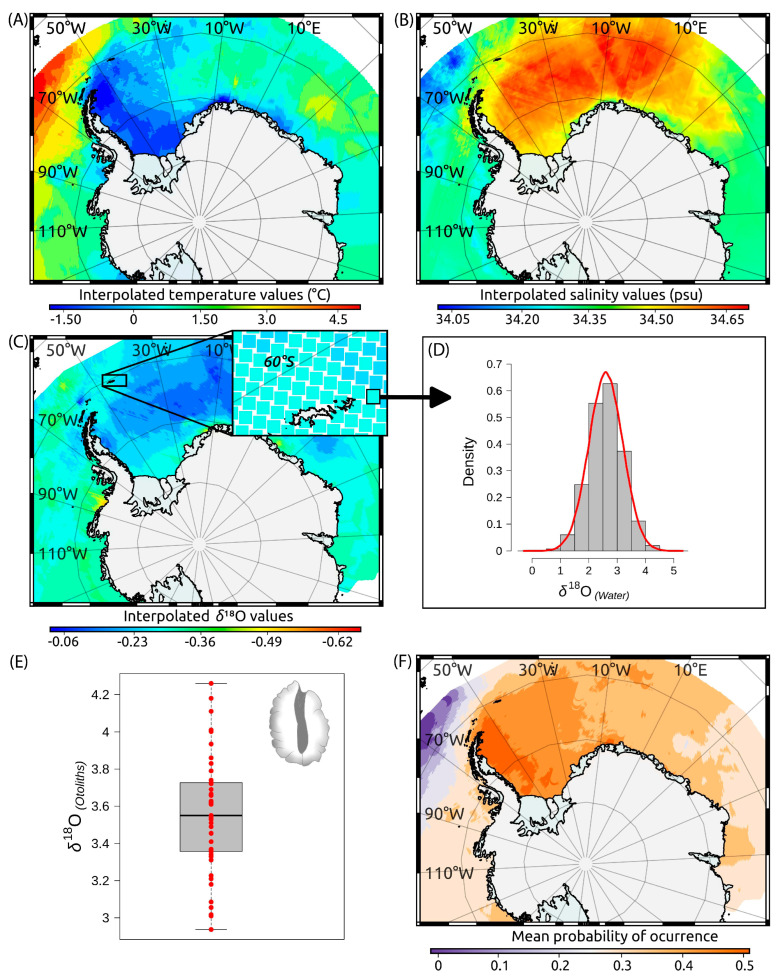
Schematic representation of the geo-location method used to identify nursery areas of *Dissostichus mawsoni* in this study. (**A**,**B**) Interpolated values of temperature and salinity in the water column, used as covariates for (**C**) kriging δ^18^O. (**D**) Probability distribution functions for expected δ^18^O values at each location and layer. (**E**) Observed δ^18^O values in sections of the sagittal otoliths of *D. mawsoni*. (**F**) Synoptic maps of averaged individual probabilities of occurrence within each grid cell.

To hindcast spatially explicit probabilities of previous occurrence for each individual across our study area [57], we compared individual δ^18^O values observed in the nuclear region of each otolith (δ^18^O_OTO1_) to the expected distribution of such values across the study area (Figure 3). To produce these expected values, we assumed otolith aragonite precipitated in equilibrium with seawater (δ^18^O_WATER_), following the empirical relationship between biogenic carbonates and δ^18^O_WATER_ derived by Thorrold et al. [58]. This equation outperformed commonly used fractionation relationships [59,60,61,62,63] as it yielded a lower mean bias (0.29‰) than other tested equations, whose mean biases ranged between −0.77‰ [59] and −1.53‰ [61]. To perform this bias testing, we compared the δ^18^O values observed in otolith edges (δ^18^O_OTO2_) to the theoretical δ^18^O_OTO2_ values predicted from the δ^18^O_WATER_ isoscapes built using each alternative equation. To do so, δ^18^O_WATER_ isoscape values (see below) were averaged over a radius of 100 km and a depth layer of 100 m around each capture location. Thus, δ^18^O_OTO2_ was predicted from δ^18^O_WATER_ as,

δ^18^O_OTO_ = 4.64 − 0.21 T °C + δ^18^O_WATER_

Since available δ^18^O_WATER_ values were reported relative to SMOW, we first converted them to VPDB following the equation,

δ^18^O_VPDB_ = 0.97006 × δ^18^O_SMOW_ − 29.94

δ^18^O_WATER_ isoscapes were built by interpolating (Bayesian kriging) all δ^18^O_WATER_ data available for the Southern Ocean in both the Global Seawater Oxygen-18 Database [64] and the World Ocean Database [65]. As in many other domains, these δ^18^O_WATER_ data were temporally and spatially limited [64]. Several studies have shown, however, that δ^18^O_WATER_ can be predicted from salinity, temperature, and, less often, from other physical variables, within oceanographic domains [66,67]. This was also the case here, where salinity, temperature, and depth explained 61% of δ^18^O_WATER_ deviance. Taking into account both the nonlinear effects of these covariates and the spatial correlation among observed δ^18^O_WATER_ values, we fit a Bayesian geo-statistical model [68] and used it to build isoscapes of predicted (interpolated) δ^18^O_WATER_ values across the whole study area, considering six depth strata, 0–50, 50–100, 100–200, 200–300, 300–400, and 400–500 m, defined to exceed the expected bathymetric range (90–400 m) for *D. mawsoni* juveniles [5,7]. World Ocean Database values of temperature and salinity were previously interpolated using the Julia package DIVAnd v2.7.12 [69]. The temporal domain used for inference about juvenile life (nuclear region of the otolith) corresponded to the yearly interval 1989–2002, set to match the putative birth years of the sampled fish. The temporal domain used for testing bias related to fractionation equations was 2011–2018, set to match the last six years of life of sampled fish (i.e., the time period represented in our edge samples).

The δ^18^O_WATER_ isoscape (mean and variance estimates) was then used to generate the probability distributions of expected δ^18^O_OTO1_ values at each location and depth stratum (Figure 3D). To accomplish this, we employed Thorrold et al.’s fractionation equation [58] and followed the same general procedure described for δ^18^O_OTO2_. Both δ^18^O_WATER_ estimation and δ^18^O_OTO_ fractionation errors were propagated at each location through a standard Montecarlo procedure with 5000 iterations. By confronting the observed δ^18^O_OTO1_ values to these expected δ^18^O_OTO1_ distributions, we ultimately estimated the individual probability of the occurrence of each fish at each location and depth stratum during its YOY stage. The individual probabilities resulting from this exercise were then averaged to produce synoptic maps within and across depth layers (Figure 3F) and to compare such probabilities between the four sub-population areas delineated by Söffker et al.’s Hypothesis 3 [29]. The log_e_-likelihood (loglik) of each location was also computed for comparative purposes as the product of all individual probabilities at each place.

## 3. Results

### 3.1. Ontogenetic Habitat Shift

**Figure 4:**
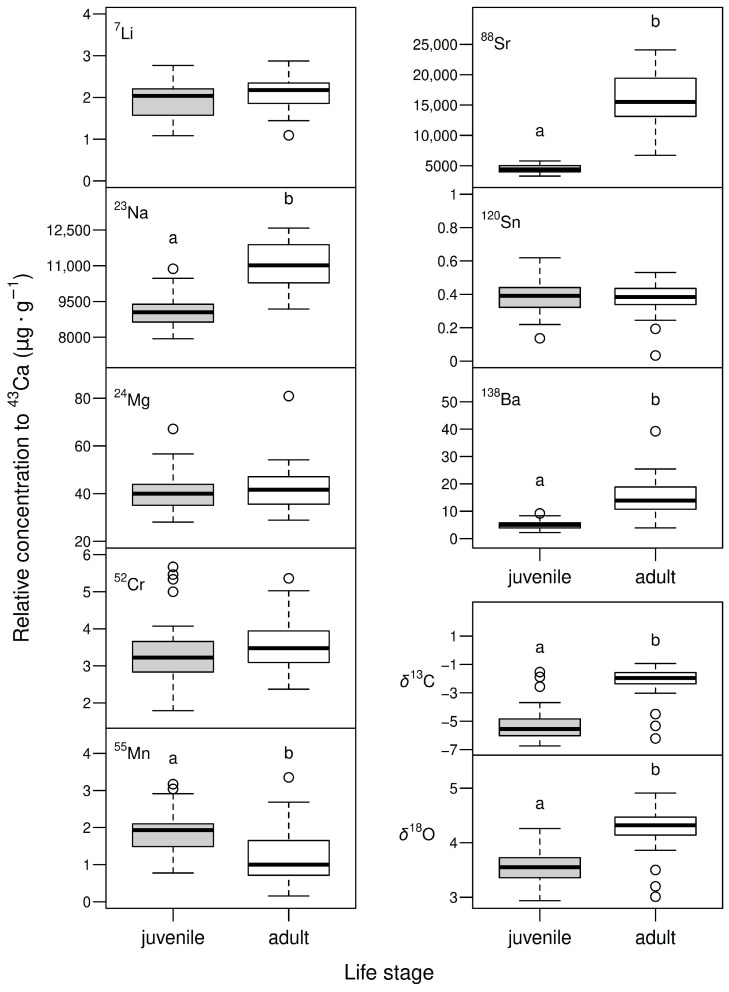
Elemental compositions (Li, Mg, Mn, Sn, Na, Cr, Sr, and Ba relative to Ca) and isotopic signatures (δ^18^O and δ^13^C) of nuclear and marginal otolith regions, representing the first year of life and the adult life stages of Antarctic toothfish (*Dissostichus mawsoni*) adults from the South Orkney Islands. Boxes represent the 25th and 75th percentiles, with whiskers extending up to 1.5 times the interquartile range or the most distant observation within this range. Different superscript letters indicate *p* < 0.05 under the null hypothesis of no difference between means.

**Figure 5:**
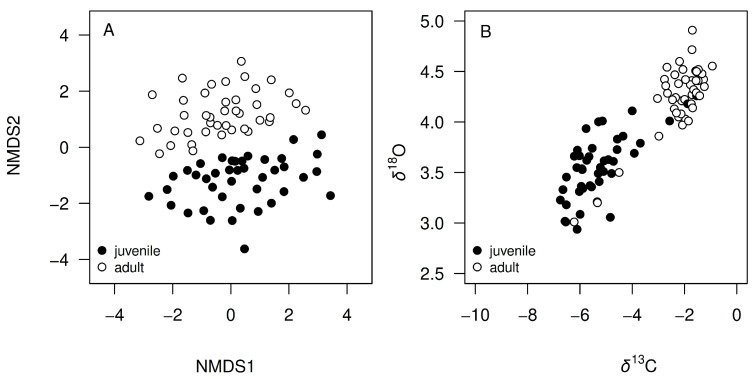
Chemical signatures observed in the nuclear and marginal regions of otoliths, representing the juvenile and adult stages of Antarctic toothfish (*Dissostichus mawsoni*) from the South Orkney Islands. (**A**) Elemental composition (Na, Mg, Mn, Sr, and Ba relative to Ca) visualized using Non-Metric Multidimensional Scaling (NMDS). (**B**) Isotopic composition of δ^13^C and δ^18^O.

The highest relative concentrations found in *D. mawsoni* otoliths corresponded to Na and Sr, followed by Ba, Cr, Li, Mn, and Sn (Table 1). The mean relative concentrations of Na, Sr, and Ba were 1.2, 3.5, and 3.0 times higher in marginal regions representing recent adult life than in nuclear regions representing juvenile (YOY) life (Table 1, Figure 4). The mean relative concentration of Mn, on the other hand, was 38% lower in the marginal region, while small differences (<10%) were found in Li, Cr, Mg, and Sn values. Multivariate analysis showed that these overall differences in elemental composition between nuclear and marginal otolith regions were large and consistent (n = 41, Pseudo-F = 10.604, *p*>F < 0.001), revealing a clear segregation between juvenile and adult signatures (Figure 5A). Large isotopic differences were also observed between otolith regions, with mean δ^18^O and δ^13^C ratios being, respectively, 20 and 58% higher in marginal regions representing adult life stages (Table 1, Figure 4). These differences between otolith regions were also evident under a bivariate analysis (n = 45, Pseudo-F = 45.245, *p*>F < 0.001, Figure 5B).

**Figure 6:**
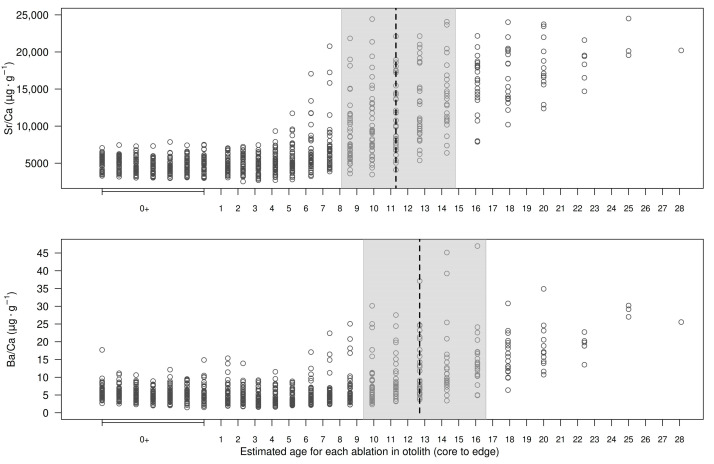
Strontium and barium profiles (relative to Ca) along the core-to-dorsal edge transect of otolith sections from Antarctic toothfish (*Dissostichus mawsoni*). The shaded area represents the estimated age range (ablations) where 50% of otoliths (P25–P75) showed the highest probability of detecting a change in element concentrations between ablations. The vertical dashed line indicates the mean age at which otoliths displayed the highest probability, based on the Bayesian change-point analysis described by Barry and Hartigan (1993) [50].

Most individual Sr:Ca profiles (~95%) showed low a probability (*p* < 0.2) of habitat shift before the tenth ablation (i.e., 4 years of life, Figure 6). Habitat shift probabilities then increased rapidly, reaching values ≥ 0.5 between ablations 15 and 19, i.e., between 8 and 15 years of life. Thus, the median age for habitat shift (*p* ≥ 0.5), estimated from Sr:Ca profiles, was equal to 11.2 years of life (Figure 6). We observed a similar pattern in Ba:Ca profiles where 75% of the fish showed low probabilities of habitat shift (<0.2) before the 14th ablation (7–8 years of life). In most fish, the Ba:Ca profiles and habitat shift probabilities exceeded 0.5 between ablations 16 and 20, equivalent to ages between 9 and 17 years of life. As a result, the median age for habitat shift (*p* ≥ 0.5) estimated from Ba:Ca profiles was equal to 12.7 years of life (Figure 6).

### 3.2. Number and Mixing Rates Between Nursery Areas

Mixtures of two sources were selected as the most informative FMD modes for both the elemental and the isotopic data sets. The second and third most informative models, corresponding to mixtures of one and three sources, exhibited much lower BIC values (ΔBIC < −12.4, Table 2). The optimal covariance models corresponded to “VVE” and “VEV” [54] for elemental and isotopic signatures. A dominant source, contributing between 73 and 86% of the mixture, and a second minor source, contributing between 14 and 27% of the mixture, were identified by the elemental and the isotopic FMD models, respectively (Table 3). The dominant source was characterized by higher Sr:Ca, Sn:Ca, and Ba:Ca, while the minor source exhibited higher values for all remaining elemental ratios, as well as for both isotopic signatures (Table 3).

### 3.3. Geo-Location of Nursery Areas

**Figure 7:**
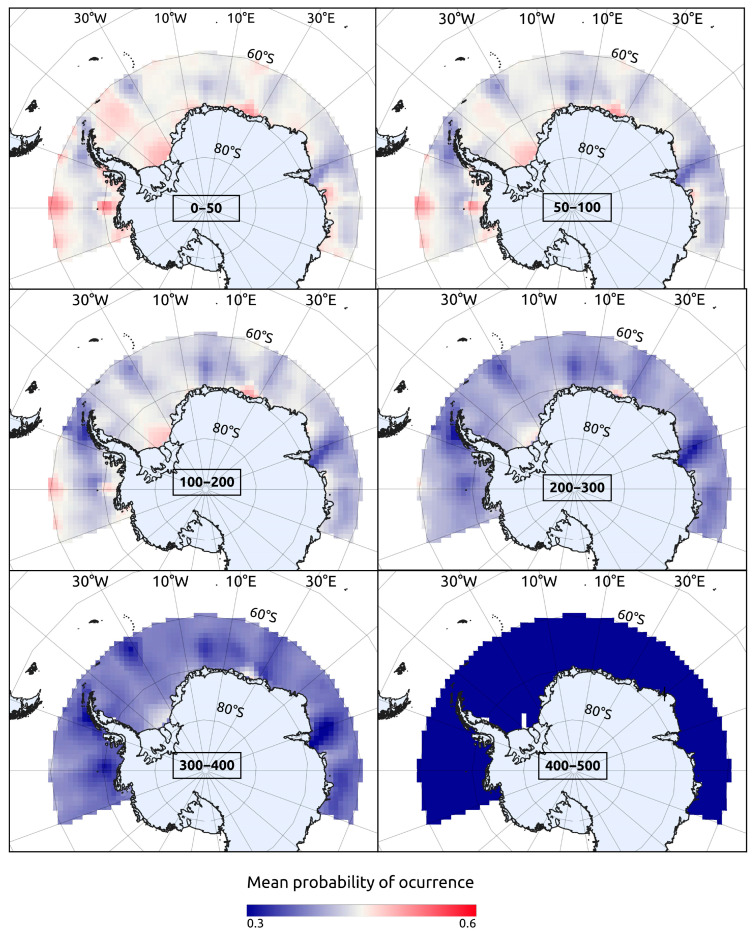
Mean probability of the occurrence of Antarctic toothfish *Dissostichus mawsoni* as young-of-the-year by location and depth stratum. Computed by comparing observed values of δ^18^O in nuclear otolith regions of adult fish to probability distribution functions estimated from seawater δ^18^O by location and depth stratum.

Probabilities of occurrence as juveniles of the year (YOY) ranged from 0.27 to 0.53, being higher in the 0–50 m (*p* = 0.45), 50–100 m (*p* = 0.43), and 100–200 m (*p* = 0.42) depth strata, decreasing with depth afterwards (*p* < 0.4). High-probability hotspots were identified in discrete coastal areas of the Bellingshausen, Wedell, Lazarev, and Cooperation seas, as well as in oceanic areas of the Bellingshausen Sea and south of the South Orkney Islands in the Wedell Sea (Figure 7).

**Figure 8:**
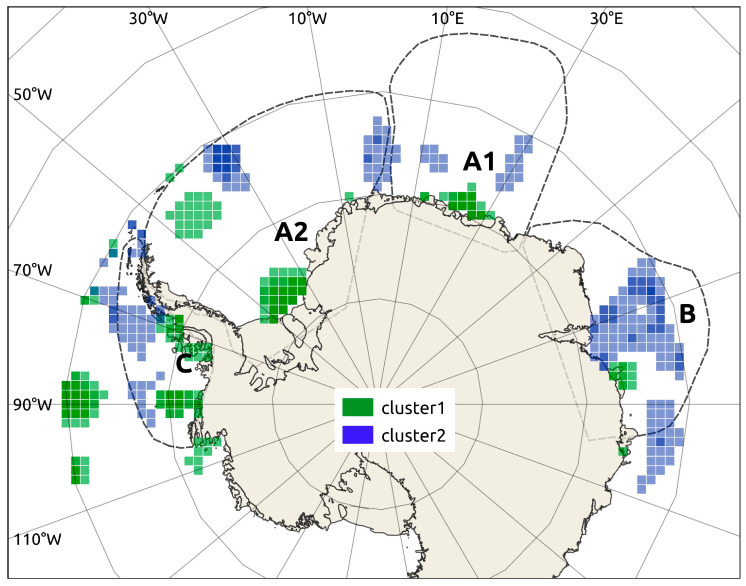
Areas with the highest probabilities (95th percentile) of origin for *Dissostichus mawsoni* adults from Source 1 (green) and Source 2 (blue), based on the most informative finite mixture distribution model (FMD). The analysis used elemental composition (Li, Mg, Mn, Sn, Na, Cr, Sr, Ba) and stable isotopes (δ^18^O, δ^13^C) in otoliths from cohorts (1989–2002) sampled around the South Orkney Islands in 2016 and 2018. Dashed lines indicate stock hypotheses (Söffker et al., 2018 [29]): A1 = Weddell sub-population 1; A2 = Weddell sub-population 2; B = Indian Ocean population; C = Bellingshausen population.

Probabilities of occurrence as YOY, averaged by location between 0 and 200 m, were similar among the four sub-population areas depicted by Söffker et al. [29]’s Hypothesis 3 (Figure 8). Probabilities averaged between 0 and 200 m ranged from 0.42 (loglik = −35.54) in area B (Cooperation Sea) to 0.44 in areas A1 (loglik = −34.33, Lazarev Sea) and A2 (loglik = −33.77, Wedell Sea). The geo-location of fish clustered by the unknown-source FMD model indicated a dispersed distribution of both clusters (sources) across the study area, with their presence in all four Söffker areas (Figure 8).

The highest 5% probability of occurrence of Cluster 1 was widely distributed across coastal regions of areas A1, A2, and C, as well as south of the South Orkney Islands (area A2) and in oceanic areas of the Eastern Pacific section (Figure 8). The highest 5% probability of occurrence of Cluster 2 tended to occupy less coastal zones and to be more dispersedly distributed than Cluster 1 across all four areas (Figure 8).

## 4. Discussion

By analysing the variability in the elemental and isotopic compositions of the nuclear regions of otoliths, we have tried to evaluate current hypotheses regarding the stock structure of *D. mawsoni* in CCAMLR Area 48. To achieve this, we have leveraged two recent opportunities made available to sample *D. mawsoni* in this region, currently closed to commercial fishing. Given these sampling limitations and despite being based on a rather limited sample size, we believe our results are consistent enough to provide useful insights into the potential number and connectivity of nursery areas and demographic units around the South Orkney Islands. While used to test current working hypotheses about demographic units in this CCAMLR area, we hope these new insights may also help guide further research efforts on *D. mawsoni* here and elsewhere.

### 4.1. Identification and Geo-Location of Nursery Areas

We found evidence that *D. mawsoni* sampled around the South Orkney Islands corresponded to a mixture of a dominant and a secondary nursery source. The number and relative contribution of these sources seemed to be consistent with Söffker et al.’s Hypothesis 2 [29], which states that two relatively discrete sub-populations mix in this area. Nonetheless, our geo-location results provide support for Söffker et al.’s Hypothesis 1 (single mixed population) as they showed no evidence that these two origins corresponded to separate geographic areas and suggested contributions from all neighbouring sub-areas from the Cooperation Sea (Sub-area A) to the Bellingshausen Sea (Sub-area C).

Given the methodological limitations of δ^18^O geo-location and limited environmental variability, we were unable to resolve connectivity at finer spatial or temporal scales, including advection between distant spawning and local nursery areas. Minimum mass requirements forced us to integrate the whole first year of life (0–540 µm from the core), collapsing in a single value all chemical signatures related to maternal effects, several months of drifting larval life, and the first months of benthic life [7]. Since LA-ICPMS provides greater time resolution, we explored this issue by fitting FMD models to specific ablations within the first year of life, finding that a three-source FMD model was more informative than single- or two-source models for each of the first four ablations, which mainly represented maternal (ablation 1) and larval life (ablations 2–4) effects. This exploratory analysis indicated that other sub-populations may also be contributing eggs and larvae to A2 nursery grounds. A relevant finding considering simulations showed that eggs and larvae from the Bellingshausen Sea (sub-population C) may be advected NE, reaching the Scottish Sea, the South Orkney Islands, and even the South Sandwich Islands [70]. Other simulations showed that some eggs and larvae released in the Lazarev Sea (sub-population B) could also be advected into the Weddell Sea by the coastal westward return of the Weddell Gyre [70].

### 4.2. Ontogenetic Habitat Shift

The predicted concentrations of early juveniles in coastal nursery habitats located east of the Antarctic Peninsula and south of the Weddell Sea suggest a combination of seasonal adult migrations to shallower spawning grounds, which are properly located to take advantage of local advective forces, such as the Weddell Gyre [29,71]. This type of retention system has already been hypothesized for multiple species, including *D. mawsoni* in Ross Sea Sub-area 88.1, where eggs spawned at the Pacific Ridge are likely transported eastward by the Ross Gyre to shelf nursery and recruitment habitats used by most juveniles [7,25]. Nonetheless, given the prolonged cumulative duration of ~9 months for *D. mawsoni* egg and larval stages [7], this species, like other Nototheniidae, seems particularly vulnerable to advection by large-scale advective forces, like the Antarctic Circumpolar Current [25].

The distinct chemical signatures we found between nuclear and marginal otolith regions indicated large environmental differences between YOY and adult habitats, suggesting ontogenetic displacements to cooler and likely deeper habitats [59,72,73,74,75]. This finding is consistent with available knowledge about the life cycle of this species [2,7], which is described as using shallow (<150 m) shelf habitats during its first year(s) of life (<13 cm LT), then moving onto use progressively deeper and wider bathymetric ranges [2,5,8]. Our analysis of Sr:Ca and Ba:Ca profiles indicated, nonetheless, that a rather discrete habitat shift would occur around 11–12 years of life, which may be related to the onset of sexual maturity [3,4] or the shift in buoyancy described by several authors to occur when *D. mawsoni* reaches a size of ~100 cm LT [5,11,12,76]. This shift in buoyancy facilitates the use of a wider bathymetric range, exploring deeper layers either for food or to avoid predation by mammals [13,77].

Differences we found in δ^13^C between nuclear and marginal otolith regions were not consistent with the expected bathymetric reduction in water DIC-δ^13^C due to variability in organic matter production/oxidation ratios [78,79] and deserve further investigation about potential dietary source and metabolic effects [58,80,81]. While YOY might rely heavily on epipelagic prey such as Antarctic krill (*Euphausia superba*) and fish larvae, adults would exploit mesopelagic cephalopods, such as Teuthidae, and fishes, such as Macrouridae, Muranolepididae, and Nototheniidae [82,83,84,85], all exhibiting greater trophic positions and, therefore, enriched δ^13^C signatures [86].

## 5. Conclusions

Overall, our results failed to provide any strong support to Söffker et al.’s hypotheses 2 or 3 [29], which assume that the South Orkney Islands stock belongs to a relatively discrete demographic unit (sub-population A2), distributed across the Weddell Sea basin. Our results show evidence that some adults sampled originated at rather distant nursery areas, including the Lazarev and the Bellingshausen seas (sub-populations B and C, respectively) areas. This apparent connectivity between potential YOY nursery grounds and feeding adult habitats also fails to provide any direct support to the philopatry hypotheses, although direct testing of this would require sampling both early larvae and spawning adults.

Our results confirm that the Southern Ocean and *D. mawsoni* otoliths exhibit enough variability to facilitate the application of otolith-based geochemical methods for investigating habitat use and connectivity patterns [74,87,88]. Nonetheless, the strength of our conclusions was affected by the limited sample size, as well as by the temporal, spatial, and vertical limitations of available δ^18^O_WATER_ data. We expect, however, that this work may stimulate additional efforts, clearly needed to clarify the demographic structure of this species in the Southern Ocean and, particularly, in CCAMLR Area 48. Within the most relevant needs, we suggest focusing on (i) revisiting connectivity patterns after collecting otolith chemistry data from adults and, ideally, juveniles sampled at each of the four sub-population areas; (ii) testing philopatry and spawning site fidelity hypotheses; (iii) assessing connectivity between spawning and nursery grounds; and (iv) ground-truthing the spatial distribution of A2 nursery grounds and assessing its potential value for other demographic units such as A1 and B. We recommend addressing these research needs before reopening the South Orkney Islands and other areas currently closed to regular fishing operations.

## Figures and Tables

**Table 1:** Estimated means and standard deviations for relative concentrations of selected elements (μmol·μmol Ca^−1^) and stable isotopes (δ^18^O and δ^13^C) in nuclear and marginal regions representing juvenile (YOY) and adult life stages of the Antarctic toothfish (*Dissostichus mawsoni*). Paired differences between stages were computed within individuals (n = 42 and 45 for elemental and isotopic signatures, respectively), fitted through a univariate linear mixed model, and tested (H_0_: intercept = 0) using a *t*-test.

Elemental Concentrations (μmol·μmol Ca^−1^) and Stable Isotopic Ratios (‰)	Life Stage				Paired *t*-Test Results		
	Juvenile		Adult				
	Mean	SD	Mean	SD	t	df	pr > t
Li	1.9	0.4	2.1	0.4	−3.21	25	<0.01
Na	9057.3	675.1	11,026.6	928.8	−11.18	25	<0.001
Mg	42.6	15.5	43.9	13.8	−0.64	23	0.53
Cr	3.3	0.9	3.8	1.1	−3.86	23	<0.001
Mn	1.9	0.5	1.2	0.710	5.30	23	<0.001
Sr	4461.9	680.9	15,683.1	4385.2	−21.32	23	<0.001
Sn	0.4	0.1	0.4	0.1	0.71	23	0.48
Ba	4.9	1.5	15.0	7.2	−8.74	23	<0.001
δ^18^O	3.6	0.3	4.3	0.3	−7.50	28	<0.001
δ^13^C	−5.3	1.1	−2.2	1.0	−10.49	28	<0.001

**Table 2:** Bayesian Information Criteria (BIC) for the three most informative unsupervised mixture models applied to elemental and isotopic signatures observed in the nuclear regions of otoliths from *Dissostichus mawsoni* adults, representing cohorts from 1989 to 2002 and sampled around the South Orkney Islands in 2016 and 2018.

Number of Sources in the Mixture	Elemental Signatures		Isotopic Signatures	
	BIC	ΔBIC	BIC	ΔBIC
1	−755.0	15.3	−208.5	8.1
2	−740.3	0	−200.4	0
3	−751.1	11.2	−208.3	7.9

**Table 3:** Nursery-source contributions and their corresponding chemical signatures in the nuclear regions of otoliths, estimated for *Dissostichus mawsoni* adults from cohorts spanning 1989 to 2002 and sampled around the South Orkney Islands in 2016 and 2018.

	Mean ± SE	
	Source 1	Source 2
Proportional contribution		
Elemental signatures (n = 37)	0.74 ± 0.061	0.26 ± 0.061
Stable isotope signatures (n = 39)	0.92 ± 0.035	0.08 ± 0.035
Elemental signature		
Li:Ca	1.66 ± 0.071	2.33 ± 0.047
Na:Ca	8912 ± 175	8926 ± 105
Mg:Ca	44.59 ± 3.523	42.64 ± 1.191
Cr:Ca	3.41 ± 0.185	2.98 ± 0.084
Mn:Ca	1.5 ± 0.093	1.98 ± 0.087
Sr:Ca	5241 ± 125	4469 ± 124
Sn:Ca	0.44 ± 0.019	0.34 ± 0.013
Ba:Ca	6.01 ± 0.262	4.08 ± 0.205
Isotopic signature		
δ^18^O	3.51 ± 0.050	4.15 ± 0.013
δ^13^C	−5.51 ± 0.130	−2.00 ± 0.052

## Data Availability

The data on the elemental and isotopic composition of otolith sections is available at DOI: 10.6084/m9.figshare.27850272.
